# Adherence of head and neck squamous cell carcinoma patients to tumor board recommendations

**DOI:** 10.1002/cam4.3097

**Published:** 2020-05-30

**Authors:** Mahalakshmi S. Rangabashyam, Shi Yan Lee, Sher Yin Tan, Stefan Mueller, Rehena Sultana, Johnatton Ho, Thakshayeni Skanthakumar, Ngian Chye Tan, Hiang Khoon Tan, Khee Chee Soo, N. Gopalakrishna Iyer

**Affiliations:** ^1^ Department of Head & Neck Surgery National Cancer Centre Singapore Singapore; ^2^ SingHealth Duke‐NUS Head and Neck Centre Singapore; ^3^ Centre for Quantitative Medicine Duke‐NUS Medical School Singapore; ^4^ Duke‐NUS Medical School Singapore

**Keywords:** adherence, compliance, Multidisciplinary team (MDT), outcomes, survival, therapy recommendations

## Abstract

**Background:**

Multidisciplinary team (MDT) meetings or tumor boards (TBs) are fundamental components of cancer treatment. Although their primary function is improved outcomes, this aspect is often underreported. The main objective of this study was to analyze the outcomes of patients with head and neck squamous cell carcinoma (HNSCC) discussed at TBs, and to compare the effect of adherence and nonadherence to recommended treatment plans on outcomes.

**Methods:**

Retrospective data analysis was conducted of HNSCC patients those who were adherent and nonadherent to TB therapy recommendations during 2008‐2009 at a comprehensive cancer center. Fisher's exact test and *t* test were used for group‐wise comparison, and Kaplan‐Meier and logistic regression models, for survival analysis and determination of the contributing factors to nonadherence.

**Results:**

Comprehensive Treatment plans were recommended by TBs in 293 HNSCC patients with curative intent. Seventy‐two patients were excluded based on the selection criteria. Among the remaining 221 patients, 172 (77.9%) were adherent to TB recommendations, while 49 (22.1%) failed to comply. Patient (n = 36; 73.5%), clinician (n = 2; 4.1%), and disease‐related (n = 11; 22.4%) factors were significant contributors to nonadherence. Mean (±standard deviation (SD)) survival time was 55.6 ± 2.32 and 29.1 ± 4 months in the adherent and nonadherent groups, (*P* < .0001, respectively). Multivariate analyses showed that gender, ethnicity, higher T‐stage, and multimodal treatment were associated with nonadherence.

**Conclusion:**

Adherence to TB recommendations improved overall survival, reflecting the importance of interdisciplinary expertise in contemporary cancer treatment. Early identification and intervention is crucial in “at risk” patients to prevent subsequent drop‐out from optimal cancer care.

## Graphical abstract

Despite the pivotal role that MDT TBs play in oncology care, the implementation of this science remains nebulous and has scope to improve. The need for standardising TB data reporting will enable to better understand HNC trends globally/regionally. Last but not the least, outcomes in HNC patients discussed at TBs and adherence of HNC patients to TB treatment plan is underreported, and analysis of this key area will be an essential cog in HNC patient management.

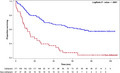

## 1 INTRODUCTION

Tumor boards (TBs) integrate oncology care through multidisciplinary team (MDT) meetings.^1^, ^2^ Given the challenge of balancing cure with quality of life, a priori multidisciplinary approach is a critical component to ensure holistic treatment planning.^1^, ^3^ Moreover, MDT meetings avoid inadvertent mono‐disciplinary bias, as a range of different specialists are involved in the decision‐making process, who collectively encompass all aspects of the diagnosis and treatment. This is especially relevant in cases where existing cancer guidelines (eg, from National Comprehensive Cancer Network) are ambiguous, or suggest divergent clinical pathways.^2^, ^4^ TBs also enable audit of clinical management, and evaluation of patient outcomes in newly diagnosed patients with head and neck squamous cell carcinoma (HNSCC), additionally providing a platform to establish databases for audit and research purposes.^4^

Several studies have focused on various facets of TB functions: how these operate, data management, challenges of implementing meetings, and importantly, on its impact on improved diagnosis and treatment.^3^, ^4^, ^5^, ^6^, ^7^, ^8^, ^9^ As a consequence, more patients would be directed to multimodal treatment, with shorter wait times to treatment commencement and overall improved coordinated care through an MDT approach.^5^, ^8^, ^10^, ^11^, ^12^ Surprisingly, a systematic review by Croke et al concluded that MDT treatment recommendations had no significant impact on patient outcomes.^13^ Similarly, another review of 27 studies reported there was no supporting evidence for relationship between MDT discussions and improved overall survival.^5^ However, both reviews included combined studies of multiple cancer types.

Based on these, we posit that patient adherence or compliance with MDT recommendations may be confounders in the impact of TB decisions on patient outcomes. It would be logical to assume that adherence to best clinical practice should have a positive impact on outcomes. However, since the onus is on the primary physician to relay MDT recommendations, and the patients ultimately make the final decision, both steps can introduce significant biases in implementation of these recommendations. Therefore, the main objective of this study was to evaluate the impact of adherence to Head and Neck Tumor Board (HNTB) treatment recommendations vs nonadherence on patient outcomes, and elucidate contributing factors that predict for nonadherence.

## 2 MATERIALS AND METHODS

### 2.1 Study design

Retrospective analysis was conducted of all HNSCC patients who were presented at TBs for 2 consecutive years (2008‐09) at a comprehensive cancer center. The study was approved by the Centralised Institutional Review Board. The data that support the findings of this study are available on request from the corresponding author. The data are not publicly available due to privacy or ethical restrictions.

### 2.2 Head and neck tumor board

At our institute, weekly HNTB meetings have been ongoing since 2000. These are attended by a range of specialists from surgical, medical, and radiation oncology, radiology, pathology, nuclear medicine, and allied health professionals. The Board discusses relevant investigations and diagnostic results, and recommends individualized treatment plan after review of all results and finalization of disease stage. Members formally document HNTB recommendations and decisions for each case. Cases include patients with newly diagnosed HNSCC and other head and neck primaries (non‐SCC). In this study, the latter were excluded. Therapeutic recommendations of MDT are as follows: (a) surgery only; (b) surgery with adjuvant treatment (radiation or chemo‐radiation therapy); (c) concurrent chemotherapy and radiation therapy; (d) neo‐adjuvant treatment (radiation or chemo‐radiation therapy) followed by surgery; (e) palliative treatment. Some of these decisions are made when postoperative histopathology is presented at HNTBs.

### 2.3 Patients

**Figure 1:**
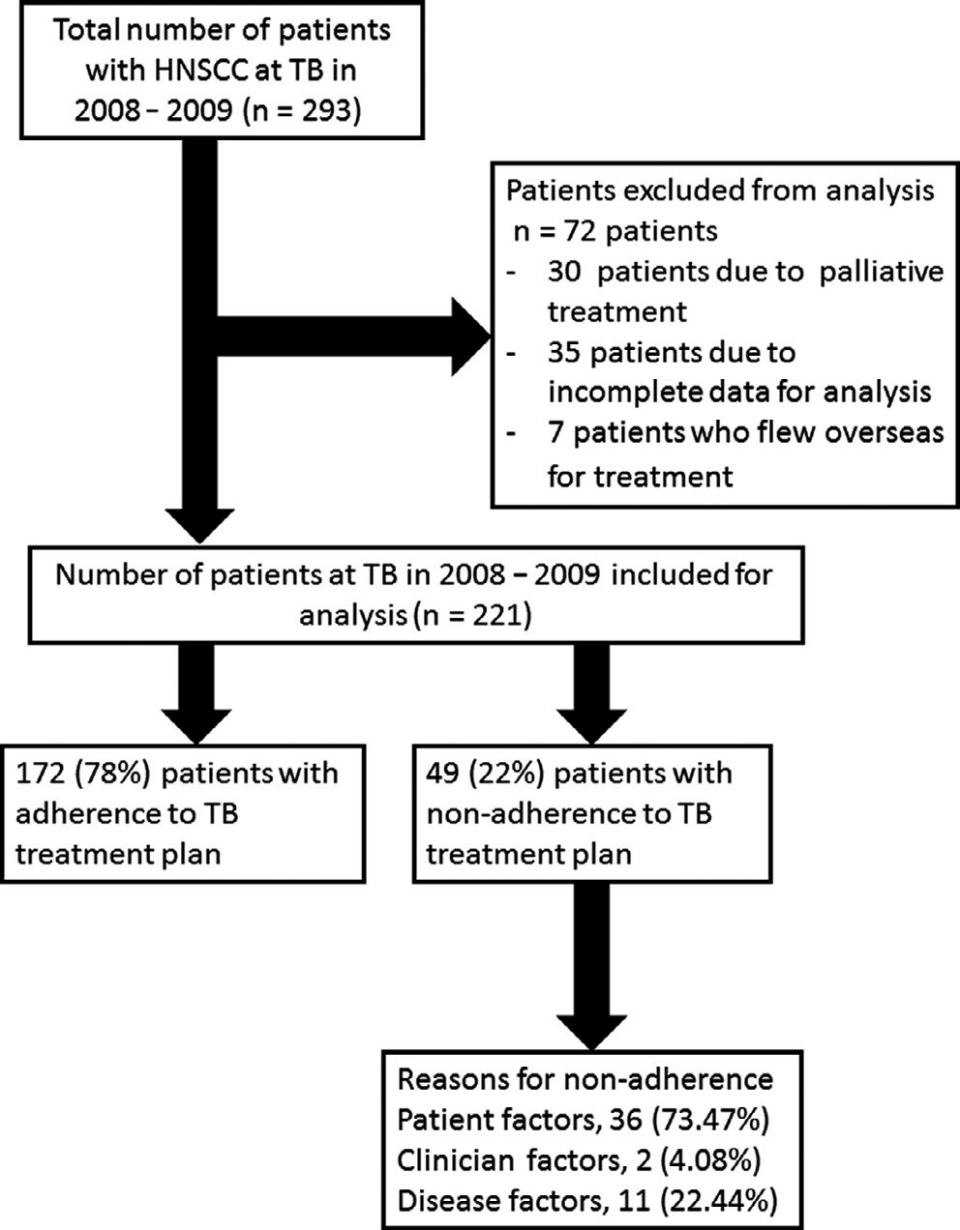
Flow‐chart of patient selection

Inclusion criteria: all HNSCC patients discussed at TBs from January 2008 to December 2009 who received therapy recommendations for curative intent. Exclusion criteria were patients presenting with incurable disease (stage IVb or IVc), recommended for palliative treatment, and in addition patients with incomplete data, including those lost to follow‐up after treatment (Figure 1). Patients with non‐HNSCC (eg, sarcomas and recurrent thyroid cancer) were also excluded.

### 2.4 Data collection

For assessment of adherence, case notes were retrieved and concordance checked between HNTB recommendations and electronic medical records to confirm whether implementation and completion of actual treatment was in accordance with HNTB decision.

### 2.5 Definition of adherence

Defining the term adherence or compliance is essential to a structured study.^14^ In our study, implementation of TB‐recommended treatment was considered for 1 year after HNTB decision based on intention to treat. Although adherence and compliance have been described interchangeably, we focused on the concept of adherence to prevent ambiguities in submission or yield from use of compliance.^15^ Moreover, adherence implies equal participation and contribution in a doctor‐patient relationship as compared with compliance, and is preferred by investigators.^16^

### 2.6 Adherence/Compliance

Data of cases with complete adherence to recommended treatments outlined during HNTB meeting, with special emphasis on entirety of adherence were collected. Patients whose clinical management conformed to all modalities of treatment recommendation were classified as the adherent group.

### 2.7 Nonadherence/Noncompliance

Data of cases with partial or complete deviation from HNTB treatment plan were collected. Patients who were noncompliant/nonadherent to HNTB decision were classified as the comparison group; those with omission of any one treatment modality in actual clinical management (eg, underwent surgery but declined chemotherapy or radiation therapy) were deemed as nonadherent or noncompliant. Special cases of patients who developed intolerance to chemotherapy after undergoing one or two cycles were classified in the adherent group.

### 2.8 Factors of HNTB treatment deviation/nonadherence

#### 2.8.1 Patient factors

Recommended treatment declined partially or completely due to any of the following reasons: fear of treatment side‐effects, expressed preference for other forms of alternative treatment, and family members declining treatment in patients with advanced age.

#### 2.8.2 Clinician factors

Attending clinician's decision to withhold adjuvant treatment after complete resection of the primary tumor.

#### 2.8.3 Disease factors

Underlying health condition that prevented complete implementation of recommended treatment (eg, comorbidities or ongoing treatment for other malignancies that compromised the ability to undergo additional treatment) (Table 2).

### 2.9 Statistical analysis

Primary outcome of adherence status was treated as binary data with categories as *“adherent*” and “*nonadherent,*” and all demographic, clinical, and treatment‐related data were summarized under adherence status. Categorical variables are summarized as frequency (percentage), and continuous variables, as mean (±standard deviation (SD)) or median (interquartile range (IQR)), whichever appropriate. Differences between the two adherence groups were compared using independent two‐sample t‐test and Chi‐square test for continuous and categorical variables, respectively. Kaplan‐Meier plots were used for overall survival, and log‐rank test was used for comparison of survival between the two adherence groups. Univariate and multivariate logistic regression analyses were performed to determine the associated risk factors for nonadherence. Quantitative association from logistic regression is expressed as odds ratio (OR) with 95% confidence interval (CI). Variables with *P*‐value < .3 in univariate logistic regression analysis were chosen for multivariable model. Multivariable model was finalized using stepwise variable selection method. Area under the curve (AUC) from receiver operating curve (ROC) analysis is reported. A value of *P* < .05 was considered as statistically significant. In this study, all tests were two‐sided, and SAS Version 9.4. (2014, SAS Institute Inc) was used for analyses.

## 3 RESULTS

A total of 293 HNSCC patients were recommended individualized TB treatment plans during the study period; of these, 72 patients were excluded for various reasons (Figure 1), and the remaining 221 patients were assessed. The cohort comprised of 172 (78%) males with mean age (standard deviation) of 62 (12.5) years. One hundred and ninety‐one (86.0%) patients presented with de novo primary SCC, while the remaining 30 (14.6%) patients, with recurrent disease (Table 1). The median (IQR) follow‐up time was 30.0 (11.3‐73.8) months.

**Table 1:** Patients’ characteristics according to treatment adherence status

Characteristics	Adherent (n = 172)	Nonadherent (n = 49)	Total (n = 221)	*P*‐value
Age of diagnosis (y), mean (±*SD*)	61.3 (± 12.3)	64.2 (± 13.2)	62.0 (± 12.5)	.1701
Sex, n (%)				.0579
Male	129 (75.0)	43 (87.8)	172 (77.8)	
Female	43 (25.0)	6 (12.2)	49 (22.2)	
Race, n (%)				.0428
Chinese	126 (73.3)	36 (73.5)	162 (73.3)	
Malay	4 (2.3)	4 (8.2)	8 (3.6)	
Indian	12 (7.0)	6 (12.2)	18 (8.1)	
Others	30 (17.4)	3 (6.1)	33 (14.9)	
Prior malignancy, n (%)				.7323
No	159 (92.4)	46 (93.9)	205 (92.8)	
Yes	13 (7.6)	3 (6.1)	16 (7.2)	
Primary/recurrence, n (%)				.1098
Primary	152 (88.4)	39 (79.6)	191 (86.4)	
Recurrence	20 (11.6)	10 (20.4)	30 (13.6)	
Subsite of cancer, n (%)				.0829
Oral cavity	77 (44.8)	24 (49.0)	101 (45.7)	
Oropharynx	18 (10.5)	2 (4.1)	20 (9.0)	
Hypopharynx	11 (6.4)	2 (4.1)	13 (5.9)	
Larynx	57 (33.1)	15 (30.6)	72 (32.6)	
Nasal cavity	3 (1.7)	5 (10.2)	8 (3.6)	
Skin	4 (2.3)	0 (0.0)	4 (1.8)	
Met. SCC of UO	2 (1.2)	1 (2.0)	3 (1.4)	
Clinical stage T, n (%)				<.0001
0	4 (2.4)	2 (4.1)	6 (2.8)	
1	50 (29.4)	4 (8.2)	54 (24.8)	
2	55 (32.4)	6 (12.2)	61 (28.0)	
3	24 (14.1)	6 (12.2)	30 (13.8)	
4	37 (21.8)	30 (61.2)	67 (30.7)	
Clinical stage N, n (%)				.5188
0	106 (62.4)	25 (51.0)	131 (60.1)	
1	16 (9.4)	4 (8.2)	20 (9.2)	
2	46 (27.1)	19 (38.8)	65 (29.8)	
3	1 (0.6)	0 (0.0)	1 (0.5)	
4	1 (0.6)	0 (0.0)	1 (0.5)	
AJCC (7th Edition), n (%)				.0003
0	3 (1.8)	0 (0.0)	3 (1.4)	
1	40 (23.7)	4 (8.2)	44 (20.4)	
2	31 (18.3)	2 (4.1)	33 (15.3)	
3	28 (16.6)	5 (10.2)	33 (15.3)	
4	67 (39.6)	36 (77.6)	103 (47.7)	
Histological grade, n (%)				.8024
Poorly differentiated	29 (17.2)	8 (16.3)	37 (17.1)	
Moderately differentiated	82 (48.5)	26 (53.1)	108 (49.8)	
Well‐differentiated	35 (20.7)	7 (14.3)	42 (19.4)	
SCC‐not otherwise specified (NOS)	23 (13.6)	7 (14.3)	30 (13.8)	
Tumor board recommendation, n (%)				.0010
Adjuvant/Neoadjuvant and surgery	47 (27.3)	27 (55.1)	74 (33.5)	
Surgery	55 (32.0)	12 (24.5)	67 (30.3)	
Primary RT/Chemo/Chemo and RT	70 (40.7)	10 (20.4)	80 (36.2)	
Modality selection, n (%)				<.0001
Surgery	47 (27.3)	9 (18.4)	56 (25.3)	
Surgery and adjuvant	53 (30.8)	8 (16.3)	61 (27.6)	
No treatment	0 (0.0)	18 (36.7)	18 (8.1)	
Primary radiotherapy	39 (22.6)	9 (18.4)	48 (21.7)	
Primary Chemo‐RT	33 (19.1)	5 (10.2)	38 (17.1)	

Note

Categorical and continuous variables are compared by Chi‐square test and independent two sample *t* test, respectively.

Abbreviation: Met. SCC of UO, metastatic squamous cell carcinoma of unknown origin; RT, Radiation Therapy.

According to our specific definition, 172 (78%) patients were adherent and 49 (22%) patients were nonadherent to MDT recommendations. The reasons for nonadherence to treatment are summarized in Table 2. In 73.4% (36/49) of cases, nonadherence was due to patients declining treatment recommendations for different reasons including fear of treatment side‐effects, poor social support, and family members’ concerns about patient's advanced age. Attending clinicians’ decision to deviate from recommended treatment was noted in 4% (2/49) of patients: close follow‐up recommended after resection of a primary tumor with wide tumor‐free margins in one, and no added survival benefit of chemotherapy deemed by the medical oncologist in the other. In 22% (11/49) of patients, nonadherence was due to disease factors: comorbidities that ruled out chemotherapy in six patients, mortality before start of treatment in three patients, and disease progression (intracranial invasion and distant metastases) exceeding the scope of TB treatment plan in two patients.

**Figure 2:**
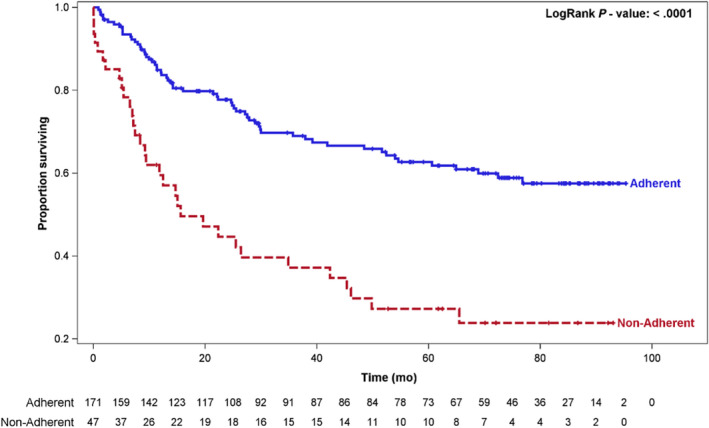
Survival chart indicating that adherence to tumor board recommendations strongly affects overall survival

Comparing clinicopathologic and treatment factors, similar characteristics were observed between the two groups (Table 1). Similar proportions of adherent vs nonadherent cases were observed in patients presenting with de novo primary and recurrent disease (88% vs 80%, and 12% vs 20%, respectively). No difference of the tumor site was observed between the groups, in which the majority of tumors originated at the oral cavity (45% vs 49%, respectively). However, 63% (109/172) of patients in the adherent group presented with early T‐stage of T1/T2, as compared to 73% (36/49) of those in the nonadherent group who presented T3/T4 disease stage (*P* = .001). No treatment was administered in approximately 40% (18/49) of nonadherent patients. Whereas, treatment plan was fully executed in half of patients in the adherent group, which comprised more than one modality of treatment modality in 50% (86/172) of patients: surgery with adjuvant treatment in 53 (30.8%) patients and chemo‐RT in 33 (19.1%) of patients (*P* = .0001). As a result, median (95%CI) overall survival of nonadherent patients was 15.6 (9.3‐42.3) months while adherent patients needed longer follow‐up time to reach median survival time (Figure 2).

**Table 2:** Factors and reasons for nonadherence

Factors for nonadherence Total N = 49 patients	Details of nonadherence
Patient factors 36 (74%) patients	Fear of side‐effects (23 patients) Declined surgery (12 patients) Declined chemotherapy (5 patients) Declined RT (5 patients) Patient declined all treatment (10 patients) Family withheld consent due to patient's old age (5 patients)
Clinician factors 2 (4%) patients	Wide margins, radiation oncologist's opinion that RT is not needed Medical oncologist's opinion that chemotherapy is not required
Disease factors 11 (22%) patients	Advanced tumor deemed nonresectable due to delay in patient's decision to comply (1 patient) Detection of distant metastasis (1 patient) Patient mortality (3 patients) Unfit for chemotherapy (6 patients)

Abbreviation: RT, Radiation Therapy.

Univariate logistic regression model showed that ethnicity, clinical T‐stage, AJCC stage, and type of treatment modality were significantly associated with nonadherence to HNTB recommendations. However, multivariate logistic regression analysis showed that (Table 3), gender, ethnicity, T‐stage, and modality of treatment were independent factors associated with nonadherence. Based on these results, female gender (OR (95%CI): 4.71 (1.45, 15.31), Malay and Indian ethnicities (OR (95%CI): 6.9 (1.0, 45.8) and 2.1 (0.7, 6.6), respectively), and advanced T‐stage (OR (95%CI): 3.4 (1.6, 7.5)) had higher odds of being nonadherent. Similarly, patients recommended for additional multimodal treatment (OR (95%CI): 3.2 (1.2, 8.1)) were less likely to adhere to HNTB treatment plans.

**Table 3:** Univariate and multivariate logistic regression analysis for associated risk factors of nonadherence

Variables	Univariate analysis		Multivariate analysis	
	Unadjusted OR (95% CI)	*P*‐value	Adjusted OR (95% CI)	*P*‐value
Age	1.02 (0.99, 1.05)	.1510		
Sex (Female vs Male)	2.39 (0.95, 6.00)	.0639	4.71 (1.45, 15.31)	.0100
Race (Reference = Chinese)		.0629+		.0168+
Indian	1.75 (0.61, 5.00)	.4059	2.1 (0.67, 6.59)	.4624
Malay	3.50 (0.83, 14.69)	.0613	6.9 (1.04, 45.81)	.0350
Others	0.35 (0.10, 1.21)	.0141	0.31 (0.09, 1.12)	.0047
Prior malignancy (Yes vs No)	0.80 (0.22, 2.92)	.7328		
Subsite of cancer (Reference = Oral Cavity)		.2677+		
Hypopharynx	0.69 (0.16, 3.05)	.6205		
Larynx	0.85 (0.41, 1.76)	.7532		
Metastatic CA of unknown primary	1.90 (0.18, 20.55)	.5310		
Nasal cavity	4.97 (1.12, 22.12)	.0206		
Oropharynx	0.43 (0.10, 1.78)	.2248		
Skin	0.35 (0.01, 9.51)	.4822		
Stage T (Stage 3/4 vs Stage 0/1/2)	5.36 (2.60, 11.06)	<.0001	3.43 (1.58, 7.46)	.0019
Stage N (Stage 3/4 vs Stage 0/1/2)	0.70 (0.02, 29.03)	.8488		
Stage AJCC7 (Stage 3/4 vs Stage 0/1/2)	5.32 (2.15, 13.21)	.0003		
Histology grade (Reference = Moderately differentiated)		.8052+		
Poorly differentiated	0.87 (0.35, 2.14)	.9509		
SCC‐not otherwise specified (NOS)	0.96 (0.37, 2.49)	.7397		
Well‐differentiated	0.63 (0.25, 1.59)	.3884		
Tumor board recommendation (Ref = Primary chemo/RT/Chemo and RT)		.0019+		.0437+
Adjuvant/Neoadjuvant and Surgery	3.89 (1.74, 8.70)	.0005	3.17 (1.24, 8.07)	.0159
Surgery	1.51 (0.62, 3.72)	.4811	1.45 (0.56, 3.73)	.6092

Note

+ represents type 3 *P*‐value.

Abbreviations: CA, Cancer; RT, Radiation Therapy; SCC, Squamous cell carcinoma.

## 4 DISCUSSION

Multidisciplinary TB meetings are considered as gold standard for treatment planning in clinical oncology. Nevertheless, only few previous studies examined implementation of TB recommendations and its impact on outcomes. To achieve our primary study goals, we determined the rate of adherence to TB recommendations and whether this impacted patient outcomes in HNSCC. Our data revealed that in approximately 80% of cases, TB recommendations were implemented, and nonadherence was mainly due to the patients’ decisions, which indicates that majority of physicians involved in patient care at our institution support the MDT/TB system. In this context, patients who did not adhere to TB treatment plans showed significantly shorter median survival, as compared to those who did (29.1 vs 55.6 months, respectively) (Figure 2). Additionally, the adherent group achieved increased overall (*P* < .001) and recurrence‐free survival (*P* = .012) compared to the nonadherent group.

Our results of adherence rates are similar to those in a German study which assessed adherence in three different TBs (head and neck cancer (HNC), sarcoma, and neuro‐oncology).^14^ In this study, it was reported that HNC TB reviewed a total of 1319 patients, among which, the recommended treatment plans were implemented in 1081 (82%) patients (partial implementation) but only in 927 (70%) patients when the same strict criterion of complete implementation as that in our study was applied. Another study included 1516 patients who were presented at brain TB in 1998‐2003; here, the authors reported that 91% of TB recommendations were fulfilled within 3 months.^17^ The higher rates in this study also likely reflects a less stringent study criteria, and a cohort which included observation for patients with benign brain tumors and non‐tumor pathologies (such as arteriovenous malformations).^17^ Nevertheless, both studies did not report the impact of adherence on outcome, and there are sparse data focused on this issue. One retrospective 12‐year analysis of patients between MDT (n = 395) vs non‐MDT (n = 331) management approach reported that the former was more likely to achieve, improved 5‐year overall survival in stage IV disease, and with greater implementation of multimodal treatment.^6^ However, the authors performed analyses on intention‐to‐treat basis, and did not consider the actual rate of implementation/adherence.^6^ Meanwhile, Kelly et al analyzed clinical factors that contributed to better outcomes in MDT‐managed patients by comparing outcomes in pre‐ and post‐MDT eras.^18^ They reported that increased adherence to multimodal treatment (66% in MDT vs 16% in non‐MDT; *P* < .0001), shorter waiting time for radiotherapy after surgery (*P* = .009), staging refinements and dental and nutritional assessments in MDT‐managed groups were significant contributing factors of superior outcome. Both these studies, however, compared patients across vastly different time periods (eg, 2001‐2006 vs 2006‐2012 for the Kelly study). In the current study, we compared outcome in patients receiving treatment during the same time period with an extended follow‐up period.

To achieve the secondary goals of our study, we determined factors leading to nonadherence to TB recommendations. Our results revealed that patient factors were the most common reason for nonadherence (73.5%; 36 of 49 patients in the nonadherent group) (Table 2), which concurs with the German study that the “patient's wish” was the main reason for deviance among HNC patients.^14^ Perceived fear of functional dysfunction or side‐effects of therapy in both physicians and patients can contribute to deviation from recommended treatment. In our cohort, almost 50% of patients in the nonadherent group expressed fear of potential side‐effects of the treatment, despite the counseling for apparent treatment benefits. Radical surgery with adjuvant therapy results in better outcomes for patients with locally advanced HNSCCs, but can also impact appearance and compromise critical functions. The ability of the physician to translate this complex concept in a balanced manner is important, for the patient to adopt TB‐recommended treatment plans. Indeed, we posit that nonadherence in a proportion of patients of minority races may reflect difficulties in conveying this message from the physician to the patient, where the nuances may be “lost in translation.” Nonadherence is a major healthcare burden without any clear solutions, studies have focused on compliance with daily medications, and their results reinforce basic principles, such as importance of patient‐doctor relationship, perception of the patient and immediate care givers on the disease, its treatment, and its impact patient's quality of life.^15^, ^19^, ^20^, ^21^ Miscommunication between the physician and patient commonly occurs in clinical oncology setting, and may culminate to nonadherence.^22^, ^23^, ^24^ Allied health teams working in concert with patients and care givers can promote better understanding and enhance adherence.^25^ A UK study revealed that failure by health providers to consider patient's perceptions and preferences is a challenge in MDT management approach which can be addressed by incorporating patient‐reported outcomes (PROs).^26^, ^27^ Based on these findings, a working group of Asian experts recommended that patient's preferences should be discussed at MDT meeting.^28^ Some oncology centers (eg, France), prefer for patients to attend TB meeting, the awareness of a team of experts to discuss the case individually and reach a consensus, could alleviate anxiety^12^; and we postulate this may promote adherence. Other studies suggested that mandatory attendance of all patients at TBs may be practically difficult or unnecessary based on evidence that presentation of the complete file of patient information eliminates the need for patients to attend TB.^14^, ^29^ In addition cancer commonly coexists with comorbidities and under estimation of comorbidities, psychosocial issues, and patient preferences can conflict with treatment implementation and interferes with established guidelines.^27^, ^30^, ^31^ Our data revealed that nonadherence of patients under cancer care was complex and multifactorial, hence early identification and intervention is the key to improved outcomes.

We obtained a low rate of nonadherence due to clinician factors (2 of 49 patients). The primary clinician in‐charge should use personal discretion to deviate from MDT treatment plan according to the clinical situation, and document reasons for deviation for medico‐legal purpose. In case of conflicting TB decisions among members, patient should be provided detailed explanation of shared decision‐making.^32^, ^33^ Members should be encouraged to express any disagreements from the majority view, which is an issue that is gaining attention since it provides scope for improvement in terms of optimization of MDT function and limits litigation.^12^

Our study has several limitations related to retrospective cohort analyses. First, relatively small study population was included; nevertheless, since our study assessed a single pathological subtype of HNSCC, the data are comparable with those of previous studies. Second, a significant number of patients (18/49) declined all forms of conventional therapy, which could skew the outcomes in the nonadherent group. These data are representative of our cultural context, where the majority of patients who declined conventional treatment sought alternative or traditional therapies. Interestingly, there is an increasing trend to alternative therapies worldwide. Third, strict criteria were used to distinguish between adherence and nonadherence as binary parameter in our study, which may not reflect real‐life situation. Regardless of these limitations, the present study indicates that implementation of TB treatment plan is a challenge, and nonadherence is inherent to patients with advanced HNSCC.

## 5 CONCLUSION

The present study highlighted TB decision‐making and implementation of recommended treatment. Identification of negative factors of implementation is the key for achieving early intervention and improving outcomes. Data collection linked to similar MDT meetings should detail the implementation of TB recommendations, and any reasons for deviation from those recommended.

## AUTHOR CONTRIBUTIONS

Mahalakshmi Rangabashyam was involved in conceptualization, data curation, investigation, methodology, project administration, supervision, validation, visualization, writing—original draft, and writing review and editing. Shi Yan Lee and Stefan Mueller were involved in conceptualization, data curation, formal analysis, project administration, resources, software, supervision, validation, and writing review. Sher Yin Tan was involved in data curation, project administration, resources, software, and writing review. Rehena Sultana was involved in data curation, formal analysis, methodology, project administration, resources, software, and writing review. Johnatton Ho was involved in data curation, formal analysis, project administration, resources, software, visualization, and writing review. Thakshayeni Skanthakumar was involved in conceptualization, data curation, formal analysis, project administration, resources, software, visualization, and writing review. Ngian Chye Tan was involved in conceptualization, project administration, validation, visualization, supervision, and writing review and editing. Hiang Khoon Tan was involved in conceptualization, project administration, resources, supervision, validation, and writing review and editing. Khee Chee Soo was involved in conceptualization, project administration, resources, supervision, validation, and writing review and editing. N. Gopalakrishna Iyer was involved in conceptualization, data curation, investigation, methodology, project administration, resources, software, supervision, validation, visualization, writing—original draft, and writing review and editing.

## STUDY DESIGN

Retrospective analysis was conducted of all HNSCC patients who were presented at TBs for two consecutive years (2008‐09) at the National Cancer Centre of Singapore (NCCS), a comprehensive cancer center. The study was approved by the SingHealth Centralised.

## Data Availability

The data that support the findings of this study are available on request from the corresponding author. The data are not publicly available due to privacy or ethical restrictions.
